# Epithelial-mesenchymal transition-related gene signature for prognosis of lung squamous cell carcinoma

**DOI:** 10.1097/MD.0000000000034271

**Published:** 2023-07-14

**Authors:** Hongmin Yu, Changxing Dai, Jie Li, Xiangning Zhang

**Affiliations:** a Department of Respiratory and Critical Care Medicine, Frist Hospital of Qinhuangdao, Hebei, China; b Otolaryngology Department, Qinhuangdao Haigang Hospital, Qinghuangdao, Hebei, China.

**Keywords:** DNA methylation, EMT, lung squamous cell carcinoma, prognostic model, survival analysis

## Abstract

Epithelial-mesenchymal transition (EMT) is associated with tumor invasion and progression, and is regulated by DNA methylation. A prognostic signature of lung squamous cell carcinoma (LUSC) with EMT-related gene data has not yet been established. In our study, we constructed a co-expression network using differentially expressed genes (DEGs) obtained from The Cancer Genome Atlas (TCGA) to identify hub genes. We conducted a correlation analysis between the differentially methylated hub genes and differentially expressed EMT-related genes to screen EMT-related differentially methylated genes (ERDMGs). Functional enrichment was performed to annotate the ERDMGs. The least absolute shrinkage and selection operator (LASSO) and stepwise Cox regression analyses were performed to build a survival prognosis prediction model. Additionally, druggability analysis was performed to predict the potential drug targets of ERDMGs. We screened 11 ERDMGs that were enriched in cell adhesion molecules and other signaling pathways. Finally, we constructed a 4-ERDMG model, which showed good ability to predict survival prognosis in the training and validation sets. The model could serve as an independent predictive factor for patients with LUSC. Additionally, our druggability analysis predicted that *CC chemokine ligand 23 (CCL23*) and *Hepatocyte nuclear factor 1b (HNF1B*) may be the underlying drug targets of LUSC. We established a new risk score (RS) system as a prognostic indicator to predict the outcome of patients with LUSC, which will help in the improvement of treatment strategies.

## 1. Introduction

Lung cancer is one of the most prevalent malignancy tumors worldwide and is the second most common cancer and the leading cause of death of cancerous patients. According to statistics, there are 2.09 million new cases and 1.76 million deaths in the world.^[1]^ Approximately 85% of lung cancer patients are diagnosed with non-small cell lung cancer (NSCLC), and of which 30% are lung squamous cell carcinoma (LUSC).^[2]^ Although significant advancements have been made in therapeutic regimens as surgery, chemotherapy, and targeted therapy, the 5-year survival of LUSC is <15%.^[3]^ Moreover, the morbidity of LUSC has increased with each passing year. Therefore, it is badly in need of finding effective drug candidates and prognostic biomarkers for LUSC.

The epithelial-mesenchymal transition (EMT) exists in various aspects of embryonic development, tissue regeneration, and organ fibrosis.^[4]^ EMT is a highly dynamic process, by which lost the polarized organization of epithelial tissues transform into mesenchymal cells with invasive capacity.^[5]^ In the process of tumorigenesis, EMT is implicated in enhancing the invasion and metastasis of cancer cells.^[6]^ Additionally, EMT is also a dynamic reversible process, and studies have reported that the phenotypic alterations of EMT are accompanied by changes in epigenetic.^[7]^ The hypermethylation promoter of EMT related-genes is the hallmark of a stable mesenchymal-like phenotype and predicts poor prognosis for most tumors.^[8]^ It is reported that the CpG island of *Grainyhead-like-2*, the inhibitor of EMT, methylation results in metastatic colonization in breast and prostate cancer.^[9]^ Vitro experiments confirm that DNA methylation inhibits the transcription of key EMT genes, such as Snail and Slug gene.^[10]^ However, there are few reports on the effects of EMT and DNA methylation on the prognosis of lung cancer.

In our study, we intend to explore novel clinical prognostic markers and drug targets for lung cancer. At first, we used RNA sequence data of LUSC from the Cancer Genome Atlas (TCGA) database and identified hub genes by weighted gene co-expression network analysis (WGCNA). Combined with differentially methylated genes (DMGs) and EMT genes, we established a prognostic risk model to predict the prognosis of patients with LUSC. Functional enrichment analysis was applied to investigate the biological of the above gene set. Additionally, we predicted the potential drug target of 11 genes, providing a new perspective for the treatment of LUSC. Our results provide new possibility for predicting the prognosis of clinical treatment of lung cancer.

## 2. Methods

### 2.1. Data source and processing

The RNA sequence data and clinical information of LUSC was downloaded from TCGA database (https://cancergenome.nih.gov/). In total, the expression data from 489 primary cancerous samples and 49 adjacent normal samples, and the clinicopathological information was summarized in Table 1. GSE30219 is an independent microarray lung cancer cohort was extracted from the GEO database (https://www.ncbi.nlm.nih.gov/geo/). A total of 47 LUSC samples from GSE30219 were used in the validation set. EMT related genes were retrieved from dbEMT data (http://dbemt.bioinfo-minzhao.org/), a database containing 1184 EMT-related genes with annotating and verified information.^[11]^

**Table 1:** Univariate and multivariate Cox regression analysis of clinical characteristics and prognosis index in LUSC of TCGA.

Characteristics	Patients	Univariate analysis		Multivariate analysis	
		HR (95% CI)	*P* value	HR (95% CI)	*P* value
Gender (male/female)	397/141	1.244 (0.919–1.683)	.157	1.184 (0.871–.608)	.281
Age (<=65 vs >65)	204/329	1.342 (1.022–1.76)	.034	1.43 (1.08–1.892)	.012
TNM stage (T3–T4/T1–T2)	98/440	1.611 (1.183–2.194)	.002	1.423 (0.968–2.094)	.073
Pathologic stage (III–IV/I–II)	96/438	1.52 (1.121–2.059)	.007	1.362 (0.938–1.979)	.104
Smoking history (current smoking/no smoking now)	197/330	0.736 (0.567–0.956)	.022	0.726 (0.557–0.946)	.018
Location (central/peripheral)	154/97	1.238 (0.846–1.811)	.271		
EGFR mutation (no/yes)	293/21	1.027 (0.502–2.1)	.941		
EML4-alk translocation (no/yes)	293/9	0.679 (0.168–2.752)	.588		
Radiation therapy (no/yes)	379/51	1.19 (0.775–1.829)	.426	0.838 (0.5–1.405)	.503
RS (high-risk/low-risk)	269/269	0.69 (0.534–0.892)	.005	0.654 (0.458–0.934)	.019

LUSC = Lung squamous cell carcinoma, RS = risk score, TCGA = The Cancer Genome Atlas.

EdgeR package in R (version 3.4.1) was used to identify the differentially expressed genes (DEGs) between LUSC and adjacent normal samples.^[12]^ DEGs were calculated using the entire transcriptome data, and p values were corrected to FDR by benjamini-Hochberg method. The cutoff value was set as |log_2_FC| > 1 and FDR < 0.05. We used ggplot2packages in R to visualize the volcano plot.^[13]^

### 2.2. Differential methylation analysis

Differential methylation analysis was performed between 42 adjacent normal samples and 370 tumor samples in TCGA database. We calculated the β value to estimate the methylation level of a given CpG probe. The formula for calculating β was as follows: β value = Imeth/(Imeth + Iunmeth), where Imeth represents the methylation intensity and Iunmeth represents unmethylation intensity.^[14]^ The t-test was used to determine the DMGs and FDR < 0.05. DMGs were featured by the absolute MTBeta-MNBeta > 0.3.

### 2.3. Weighted gene co-expression network analysis

The “WGCNA” R package was implemented on constructing a co-expression network to identify the hub genes in LUSC.^[15]^ Firstly, we constructed a hierarchical clustering dendrogram of the TOM matrix to classify the similar genes expression profiles into different gene modules by the average distance with a minimum size threshold of 20. Then, the different module eigengenes (MEs) were correlated with tumor samples, and we can easily confirm MEs that are highly relevant to clinical traits. gene significance (GS) is the absolute value of the correlation between individual genes and clinically interesting trait, module membership (MM) was calculated the correlation of MEs and gene expression profiles. GS and MM are highly correlated, which prove that the elements in the modules are highly related to clinical traits.^[16]^ These genes acted as hub genes for further research.

### 2.4. Functional enrichment analysis

Gene ontology (GO) was conducted and visualized by the “ClusterProfiler” R package, and annotated the function of EMT-related genes. ClusterProfiler aims to provide statistical analysis of GO and a visualization tool for comparing functional profiles between gene clusters.^[17]^ *P* < .05 was considered statistically significant.

### 2.5. Establishment of prognostic predictive model

The univariate Cox regression analysis was implemented on 11 EMT-related differentially methylated genes (ERDMGs) to analyze the relationship between genes expression and overall survival (OS) of LUSC patients (*P* < .05). Afterward, the least absolute shrinkage and selection operator (LASSO) regression by R package was used for reduce the dimensionality of variable selection in a Cox regression.^[18]^ Multivariate logistical regression was used to develop ERDMGs related LUSC classifier.

The prognostic risk score (RS) model was calculated as follows:


$$\text{RS}=\sum_{i=1}^{n}\left(Coef_{i}*x_{i}\right)$$

n represents the gene number in the module, Coef_i_ is the regression coefficient of each gene, x_i_ is the mRNA expression level of each gene.

The LUSC patients were categorized into high- and low-risk groups by the median RS for the survival analysis. The log-rank test Kaplan–Meier survival analysis was implemented to calculate the OS in high- and low-risk group, and was plotted by “survminer” and “survival” R package. The receiver operating characteristics (ROC) curve was used to assess the specificity and sensitivity of the prognostic RS model at different endpoint (1, 3, and 5 years). Meanwhile we calculated the area under the ROC curves (AUC). In addition, in order to further investigate the prognostic effect of our prognostic model, we compered clinical characteristics (including gender, age, tumor, pathologic stage, smoking, location, EGFR mutation, EML4-alk translocation, radiation therapy, new tumor event after initial treatment, person neoplasm cancer status) with PI by univariate and multivariate Cox proportional hazards regression analyses.

### 2.6. The prediction of drug target

We predicted druggability of protein pocket of 10 EMT-related genes to find the possible drug targets using PockDrug-Server. PockDrug-Server (http://pockdrug.rpbs.univ-paris-diderot.fr/) is a website that based on the surface of potential binding cavities from amino atoms to predict the drugability of protein pockets. It uses different pocket estimation methods to clearly efficient distinguish druggable from less druggable.^[19]^ A druggability probability of more than 0.5 was considered a druggable pocket.

For drug repositioning, we constructed the protein-protein interaction (PPI) network of the proteins with drugability pockets and identified the potential targeted drug by interacting proteins in the network.^[20]^ Drug data was obtained from Drugbank (https://go.drugbank.com/). The Search Tool for the Retrieval of Interacting Genes database (http://string-db.org/) was used to analyze the interaction between the proteins,^[21]^ the minimum required interaction score is 0.7. The PPI network was generated by the Cytoscape (version 3.3.0) software.

### 2.7. Statistical analysis

All statistical analyses were used R software (version 3.6.0). A Cox proportional hazards regression method was performed to reflect the relationship of candidate EMT-related genes and OS. Pearson correlation was used to analysis the correlation between EMT genes expression and DMGs. The cutoff criteria were set as correlation coefficient > 0.7. *P* < .05 was considered statistically significant.

## 3. Results

### 3.1. Co-expression network identified key modules related to clinical traits

**Figure 1.**
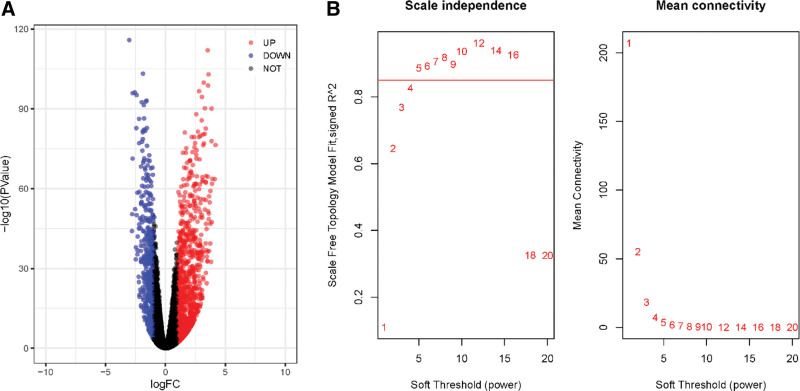
The differential expression analysis in LUSC. (A) Volcano plot of DEGs between LUSC and normal samples. X-axis is the fold change of genes expression, and y-axis is the *P* value. (B) Analysis of the soft-thresholding power in WGCNA, scale-free index (β) on the left, x-axis and y-axis represent soft-thresholding power and scale-free fit index respectively. The mean connectivity on the right, x-axis and y-axis represent soft-thresholding power and mean connectivity (degree) respectively. DEGs = differentially expressed genes, LUSC = lung squamous cell carcinoma, WGCNA = weighted gene co-expression network analysis.

**Figure 2.**
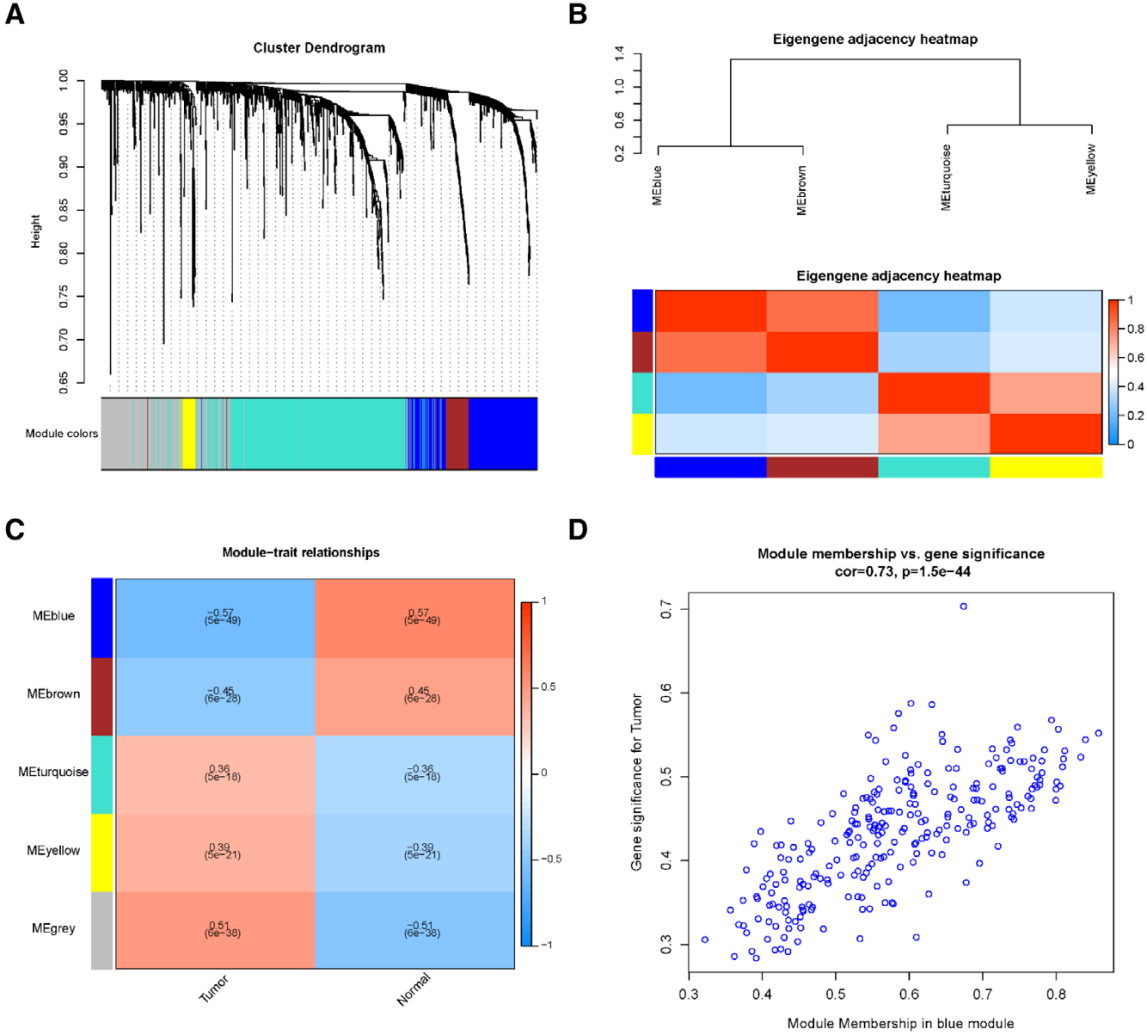
The key gene module identified by WGCNA. (A) The cluster dendrogram of co-expression network modules. Different colors represent different types of modules. (B) Hierarchical clustering of module hub genes and the eigengene adjacency heatmap of hub gene network. Red represents positive correlation and blue represents negative correlation. (C) Heatmap of the correlation between MEs and different tissue samples. Each row represents a ME, the 2 columns represent tumor and normal samples. The corresponding correlation and *P*-value were shows in each cell contain. (D) Scatter plot of gene significance (y-axis) and module membership (x-axis) in blue module. MES = module eigengenes, WGCNA = weighted gene co-expression network analysis.

The expression profiles of LUSC were obtained from TCGA database. A total of 551 samples, including 502 cancer tissue samples and 49 adjacent normal tissue samples, were subjected to differential expression analysis. Volcano plots were constructed to display the distribution of each gene according to |log_2_FC| >1 and FDR < 0.05 (Fig. 1A). Based on the differential expression analysis, 1168 DEGs were identified in LUSC samples compared to that in normal samples, with 426 upregulated and 742 downregulated genes. Subsequently, we used the DEGs to construct a co-expression network using WGCNA. The soft threshold β was set at 9 (R^2^ = 0.85) to create a scale-free network (Fig. 1B). Based on WGCNA analysis, 5 gene subsets were identified by comparing the gene expression profiles of tumor and para cancer tissues (Fig. 2A). The interactions between the 5 modules are shown in Figure 2B. We then calculated the correlation between each ME and the tumor and its adjacent tissues. As illustrated in Figure 2C, the blue module contains genes that are most differentially expressed in tumor and paracancerous tissues compared to other modules (r = −0.57, *P* = 5e-49). The scatter plots of GS and MM showed a high correlation in the blue module (cor = 0.73, *P* = 1.5e-44, Fig. 2D). Thus, the blue module was selected as being significantly related to LUSC clinically, and the hub genes in the blue module were used for further analysis.

### 3.2. Identification of EMT genes with altered DNA methylation status in LUSC

**Figure 3.**
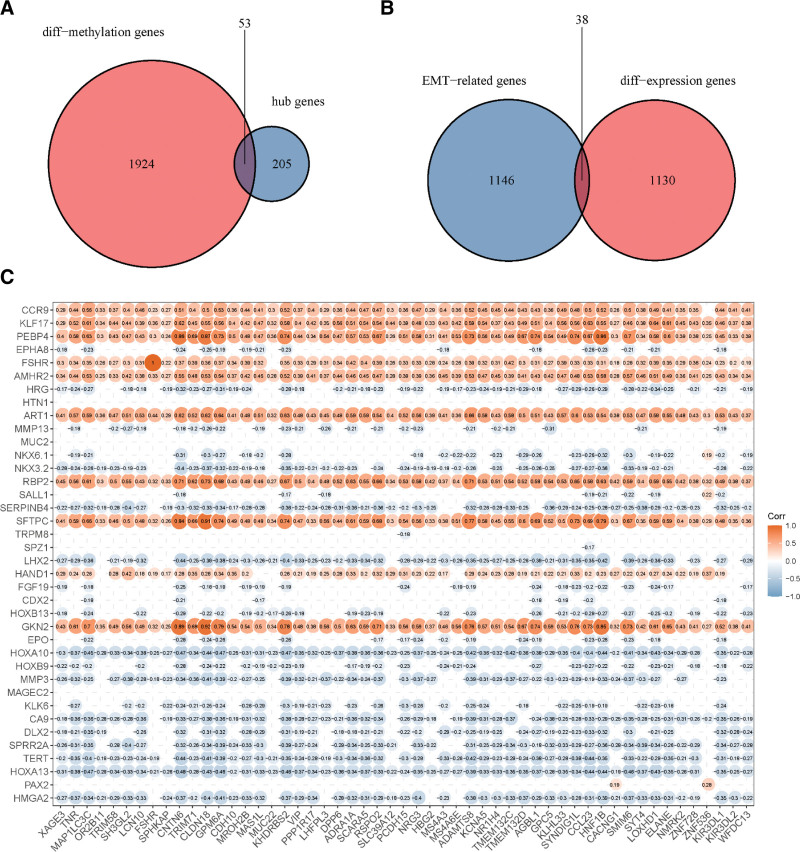
Identified genes associated with EMT genes and altered DNA methylation. (A) Venn plot of hub genes associated with DMGs. (B) Venn plot of DEGs associated with EMT genes. (C) The correlation between EMT genes and DMGs. *P* < .05 was considered statistically significant. X-axis is 53 DMGs, and y-axis is 38 EMT-related genes. DEGs = differentially expressed genes, DMGs = differentially methylated genes, EMT = epithelial-mesenchymal transition.

To identify the EMT genes with altered DNA methylation in LUSC, we explored the correlation between EMT genes and DMGs. We used DNA methylation data from TCGA database for differential methylation analysis. In total, 1977 DMGs were identified. Out of the 1977 DMGs and 258 hub genes, we identified 53 genes common in both groups (Fig. 3A). A total of 1184 EMT genes were obtained from the dbEMT data. Out of the 1184 EMT genes and 1168 DEGs, we identified 38 genes common in both groups (Fig. 3B). Subsequently, Pearson correlation was used to analyze the gene expression correlation between the 53 DMGs and 38 EMT genes. The cutoff value was set as follows: correlation coefficient > 0.7 and *P* value < .05. As shown in Figures 3C, 11 DMGs were significantly correlated with the EMT genes, which were designated as ERDMGs.

### 3.3. Identifying the prognostic signature of ERDMGs

**Figure 4.**
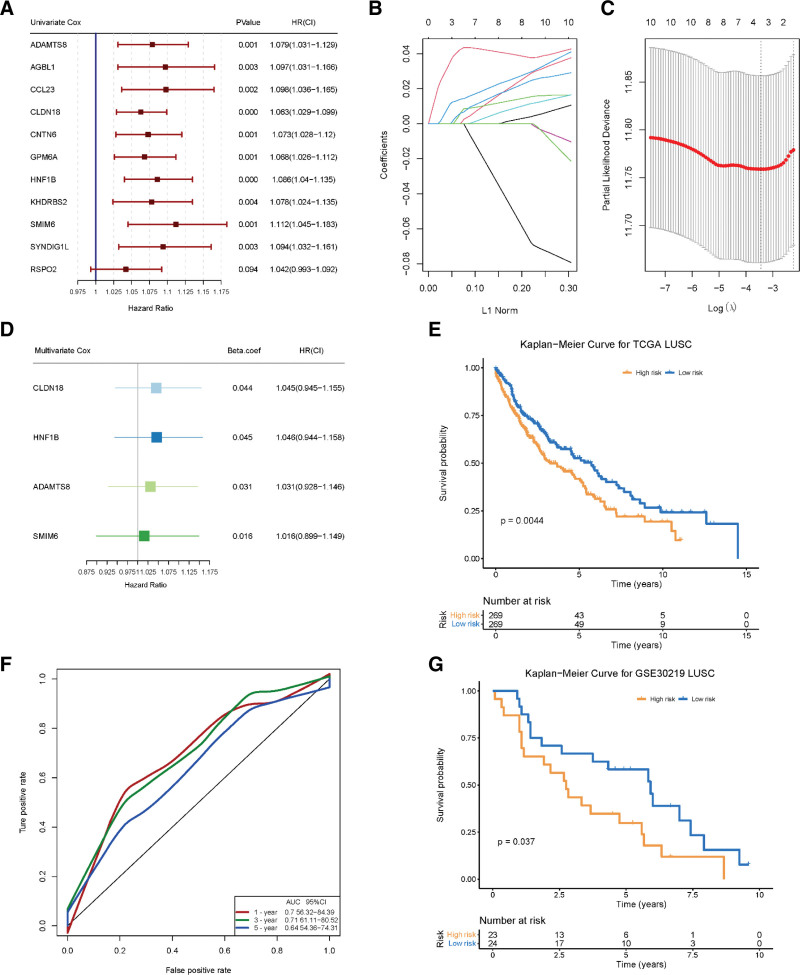
ERDMGs for constructing predictive model. (A) Forest plot for the result of univariate Cox regression analysis in 11 ERDMGs. (B) LASSO coefficient profiles of the 10 ERDMGs. (C) Partial likelihood deviance of different numbers of variables by 10-fold cross-validation for via minimum tuning parameter in the LASSO regression. The red dots represent the partial likelihood deviance values and plotted against log (λ). The first and second dotted vertical line represented optimal values by minimum criteria and 1-SE criteria. (D) Forest plot for the result of multivariate Cox regression analysis in 4 ERDMGs. (E) The survival curve for patients in training set. *P* value < .05. (F) ROC curve of training set for 1, 3, and 5 yr survival prediction. (G) The survival curve for patients in validation set. *P* value < .05. ERDMGs = EMT-related differentially methylated genes, LASSO = the least absolute shrinkage and selection operator, ROC = the receiver operating characteristics.

To investigate the prognostic role of ERDMGs in LUSC, univariate Cox proportional hazard regression analysis was combined with LASSO regression and Cox multivariate regression analyses to establish an effective predictive prognostic model. We found that 10 of the 11 ERDMGs were significantly related to OS in LUSC (*P* < .05), using univariate Cox proportional hazard regression analysis (Fig. 4A).

**Figure 5.**
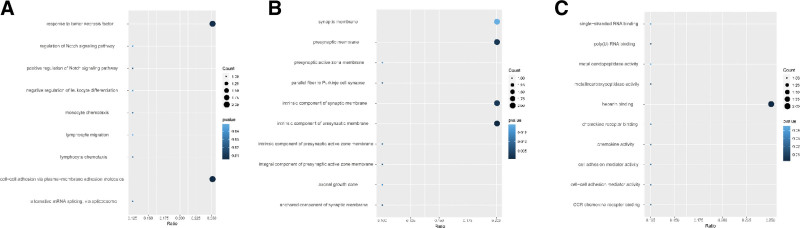
The GO analyses of 10 ERDMGs. (A) biological process, (B) cellular component, and (C) molecular function of ERDMGs. ERDMGs = EMT-related differentially methylated genes, GO = gene ontology.

The results of the GO enrichment analysis of this 10 ERDMGs are shown in Figure 5. The results revealed that the 10 ERDMGs were significantly enriched in cell−cell adhesion via plasma−membrane adhesion molecules, response to tumor necrosis factor, and the Notch signaling pathway (Fig. 5A). The cellular component category showed that the ERDMGs were significantly enriched in the intrinsic and integral component of the presynaptic active zone membrane, presynaptic and synaptic membrane (Fig. 5B). Moreover, G protein-coupled receptor and Notch binding were the representative enrichment terms in the molecular function category; the ERDMGs were also enriched in cell adhesion, which resulted in the transformation of epithelial mesenchymal cells (Fig. 5C). Notch1, which plays an important role in the Notch signaling pathway, has been reported to induce mesenchymal-to-epithelial transition, which is the inverse process of EMT.^[22]^

The LASSO regression analysis of the 10 candidate ERDMGs obtained 4 ERDMGs and the survival data of the discovery cohort (Fig. 4B and C). Multivariate Cox proportional hazard regression analysis was used to evaluate the prognostic value of the 4 ERDMGs (Fig. 4D). The survival RS was calculated using the following formula:

RS = 0.044 × *Claudin-18* expression + 0.045 × *HNF1B* expression + 0.031 × *A disintegrin and metallopeptidase with thrombospondin motif type 8* expression + 0.016 × *SMIM6* expression. (The expression of the 4 ERDMGs is listed in Supplemental Digital Content1 [see Table S1, http://links.lww.com/MD/J255, Supplemental Content, which illustrates the expression data in TCGA of 4-ERDMGs model]).

We used the LUSC data (n = 538) retrieved from TCGA as the training set and calculated the RS based on this model. The parents in the training set were classified into low- and high-risk groups based on the median RS value. The Kaplan–Meier curve showed that the survival rate of the high-risk group was significantly lower than that of the low-risk group (*P* < .05; Fig. 4E). ROC curves in the training set, with 1, 3, and 5-year AUCs being 0.7, 0.71, and 0.64, respectively, were an indicator of survival prediction with improved accuracy.

To validate the applicability and classification effect of the 4-ERDMG model, we mined the public RNA data GSE30219 from GEO as the validation set and divided the patients into high- and low-risk groups based on the median value of the RS (Fig. 4G). The survival curve showed that the OS of the low-risk group was significantly better than that of the high-risk group (*P* < .05). In addition, considering the clinical characteristics, we evaluated the OS of patients with the clinical characteristics listed in Table 1 using univariate and multivariate Cox regression analyses. 4-ERDMG model exhibited potential prognostic value in univariate and multivariate analyses. Owing to its good stability and robustness, the 4-ERDMG model could be an ideal model for predicting the prognosis of LUSC.

### 3.4. Predicting potential drug targets related to ERDMGs

**Figure 6.**
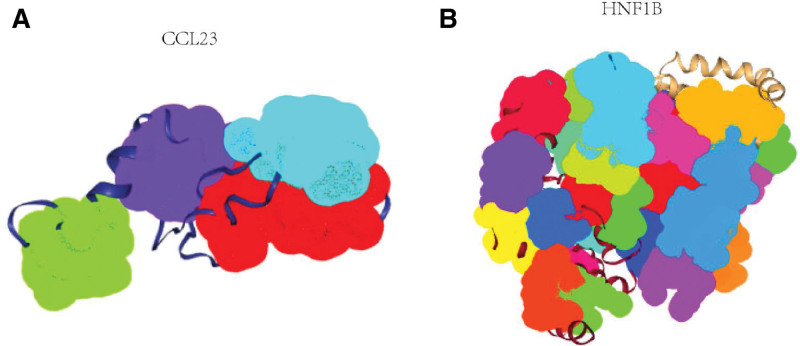
The protein pocket of CCL23 and HNF1B. CCL23 = CC chemokine ligand 23, HNF1B = hepatocyte nuclear factor 1b.

As EMT affects tumor metastasis, we aimed to identify potential drug targets related to ERDMGs for the treatment of metastatic LUSC. We used PockDrug-Server, an online tool, to predict the druggability of ERDMG protein pockets. Druggability is the capacity of a protein to bind drug-like molecules with high affinity.^[23]^ It has been proven that a pocket of a protein is druggable and may be a potential drug target.^[23]^ The results showed that CC chemokine ligand 23 (CCL23) and hepatocyte nuclear factor 1b (HNF1B) may be the underlying drug targets, which have 2 and 3 druggable protein pockets, respectively (druggability probability > 0.5; Fig. 6, Supplemental Digital Content 2 [see Table S2, http://links.lww.com/MD/J256, Supplemental Content, which illustrates Parameters of the 8 protein pockets in CCL23] and Supplemental Digital Content 3 [see Table S3, http://links.lww.com/MD/J257, Supplemental Content, which illustrates Parameters of the 8 protein pockets in HNF1B]). However, no targeted drugs for *CCL23* and *HNF1B* were identified in the Drugbank.

**Figure 7.**
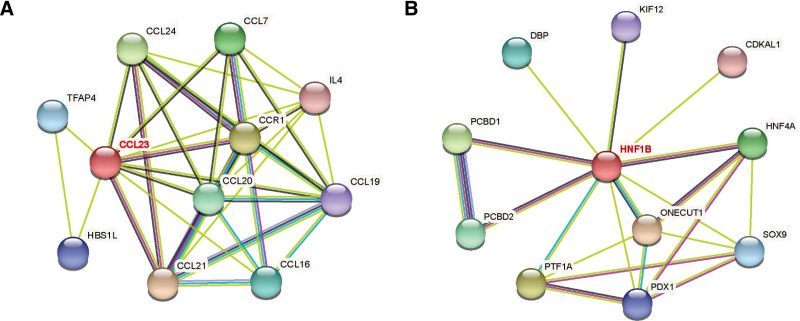
The PPI network of CCL23 and HNF1B. CCL23 = CC chemokine ligand 23, HNF1B = hepatocyte nuclear factor 1b, PPI = the protein-protein interaction.

To identify potential targeted drugs, we constructed PPI networks of CCL23 and HNF1B according to the drug repositioning theory. Drug repositioning is a method that involves the new use of approved drugs and indirectly determines the targeted drugs of key genes in tumors by targeting the interacting proteins in the PPI network.^[20,24]^ The results showed 10 nodes in the PPI network for CCL23 and HNF1B (Fig. 7), and we observed that pascolizumab targeted IL-4 in the PPI network of CCL23 and artenimol targeted PDX1 in the PPI network of HNF1B, which may be the drug targets for LUSC.

## 4. Discussion

EMT is associated with invasion and metastasis and is one of the main reasons for the poor prognosis of LUSC. Thus, we screened ERDMGs using WGCNA and differential methylation analysis and obtained a 4-ERDMG prognostic model to assess the risk of patients with LUSC. Previous studies have indicated that EMT-related gene expression can be used as a prognostic marker for LUSC. Li et al determined that downregulation of *SET binding protein 1* induces EMT to stimulate the metastasis of NSCLC cells and serves as a prognostic biomarker for NSCLC.^[25]^ Wang et al showed that *Keratin 17* promoted EMT by upregulating Snail expression, and overexpression of *Keratin 17* predicted poor prognosis in patients with NSCLC.^[26]^ Many studies have explored single EMT-related genes as prognostic markers for NSCLC; however, in this study, we found that an expression set of ERDMGs served as a prognostic biomarker and constructed a prognostic model that significantly distinguished the RSs of patients with LUSC. This expression set was related to EMT and the DMGs in LUSC. Many models for studying differential methylation exist; however, little is known about DNA methylation and EMT gene expression. Wang et al established a nine-gene prognostic methylation model for lung adenocarcinoma.^[27]^ Li et al constructed 12 aberrantly methylated gene predictive models related to OS in LUSC patients by univariate and multivariate Cox regression analyses.^[28]^ DNA methylation and EMT gene expression assess the damage to the genomes of cancer patients using epigenetics and transcriptomics, respectively. Our prognosis model considered both factors and evaluated the damage to the genome of LUSC patients from multiple perspectives. Meanwhile, to further demonstrate the value of 4-ERDMG model for the prognosis of LUSC patients, we compared it with other study. Liu et al, reported a prognosis model based on 3 glycolysis gene signature. The AUC for RS of Liu model at the 1-, 3-, 5-years OS was 0.629, 0.665, and 0.636, respectively.^[29]^ It was obviously decreased compared with our model in predicting 1-, 3-, and 5-years OS and 4-ERDMG model had much better predictive effect on the prognosis of LUSC patients (Fig. 4F). In addition, we have performed univariate and multivariate Cox regression analyses which included factors like gender, age, TNM stage, pathologic stage, smoking history, location, EGFR mutation, EML4-alk translocation, radiation therapy, and RS of 4-ERDMG model. The RS was significantly associated with OS by univariate and univariate analysis (HR = 0.69, *P* = .005, and HR = 0.654, *P* = .019). The result show 4-ERDMG model was more correlated to LUSC patients’ survival than TNM stage, pathologic stage, and smoking history (Table 1). Some of these ERDMGs have been recognized as being associated with tumorigenesis. *A disintegrin and metallopeptidase with thrombospondin motif type 8, KHDRBS2, GPM6A*, and *HNF1B* are tumor suppressor genes.^[30–32]^ *GPM6A* expression was suppressed by miR-96 to promote hepatocellular carcinoma proliferation, migration, and invasion.^[32]^ Methylation of *HNF1B* promoter inhibits its expression and promotes EMT in prostate cancer.^[33]^ Additionally, low expression of *CCL23* in hepatocellular carcinoma is not beneficial to the anti-tumor immune response and results in poor prognosis.^[34]^ It has been reported that *Claudin-18*-*ARHGAP* fusion is a molecular characteristic of gastric cancer and is related to invasion and poor prognosis.^[35]^ However, *ATP/GTP binding protein like 1* polymorphism is associated with a lower risk of lung cancer.^[36]^ Interestingly, *SYNDIG1L* and *CCL2* were correlated with excitatory synapses and neurodevelopment.^[37,38]^ This finding is consistent with the results of the GO analysis of cellular components. Some studies have demonstrated that metastatic brain cells interact with neurons.^[39]^ Compared with the clinical characteristics, the 4-ERDMG model exhibited salience. Prognostic model studies have shown that ERDMGs promote tumor progression and can serve as molecular markers to assess the prognosis of various tumors. We analyzed data from TCGA for several other cancer types. However, we found that the ERDMG model was not applicable to other cancers, such as breast cancer (*P* = .34) (see Fig. S1, http://links.lww.com/MD/J258, Supplemental Content, which is the survival curve for TCGA breast cancer patients) and pancreatic cancer (*P* = .42) (see Fig. S2, http://links.lww.com/MD/J259, Supplemental Content, which is the survival curve for TCGA pancreatic cancer patients).

Drugs targeting oncogenic signaling pathways have been reported to have an impact on the EMT status of cancer.^[40]^ To elucidate whether ERDMGs could be used as drug targets for LUSC treatment, we predicted the druggability of ERDMGs using PockDrug-Server. Druggability is the affinity of a protein that plays an important role in tumorigenesis and binds to a small-molecule drug.^[23]^ Owing to the ambiguity of the protein pocket boundary and the flexibility of the proteins, a single method is not accurate in predicting the druggability of protein pockets. The PockDrug-Server is an online tool for predicting the druggability of protein pockets. It integrates 4 methods (prox4, prox5.5, fpocket, and DoGSite) to evaluate the druggability of protein pockets in terms of the binding position of the ligand protein and the druggability prediction of apo pockets.^[19]^ Hussein et al compared the accuracy of a single method with that of PockDrug-Server in predicting the druggability of protein pockets and found that the accuracy of PockDrug-Server was 20% higher than that of a single method.^[19]^ Regad et al used PockDrug-Server to extract the inhibitor-binding site of the uPA catalytic domain, p53, and PR1.^[41]^ Our study elucidated that CCL23 and HNF1B have druggability in protein pockets. To predict the underlying target drugs of CCL23 and HNF1B, we constructed a PPI network based on drug repositioning. Drug repositioning is a method used to discover the novel uses of approved drugs through the PPI network.^[42]^ Shahjaman et al predicted 16 small-molecule drugs for the treatment of ovarian cancer by constructing a PPI network of DEGs.^[43]^ Wang et al identified 15 drugs suitable for treating melanoma by constructing a PPI network of hub genes in the co-expression network.^[44]^ Based on the PPI network of CCL23 and HNF1B, we identified that artenimol and pascolizumab may be appropriate for the treatment of patients with LUSC. Clinical studies on blocking of IL-4 in patients with pulmonary tuberculosis by pascolizumab (NCT01638520) are ongoing. However, there are no clinical studies on the efficacy and safety of artenimol and pascolizumab in the treatment of LUSC. We speculate that this may be a direction for future research on LUSC treatments.

A prediction model was established and evaluated based on the common datasets. We hope to obtain more comprehensive data in the future and to build a more robust prognostic evaluation model. In conclusion, our study provides an ERDMGs model to predict the prognosis of LUSC patients and predict possible drug targets that could alter the state of EMT in LUSC.

## 5. Conclusions

Our study proposes a novel 4-ERDMG model to predict the prognosis of patients with LUSC, and the feasibility of the proposed model was validated. The ERDMGs were enriched in leukocyte transendothelial migration and cell adhesion molecules related to tumor metastasis. In addition, *CCL23* and *HNF1B* have druggability protein pockets and may be drug targets of LUSC.

## Author contributions

**Conceptualization:** Xiangning Zhang.

**Data curation:** Hongmin Yu, Changxing Dai.

**Formal analysis:** Jie Li.

**Methodology:** Changxing Dai.

**Project administration:** Xiangning Zhang.

**Supervision:** Xiangning Zhang.

**Validation:** Changxing Dai, Jie Li.

**Visualization:** Hongmin Yu, Jie Li.

**Writing – original draft:** Hongmin Yu, Changxing Dai.

**Writing – review & editing:** Xiangning Zhang.

## Supplementary Material










