# Supplementary material for: Epithelial-mesenchymal transition-related gene signature for prognosis of lung squamous cell carcinoma
Source: Medicine (Baltimore). 2023 Jul 14;102(28):e34271. doi: 10.1097/MD.0000000000034271 (PMC10344514; doi:10.1097/MD.0000000000034271)

Supplemental Digital Content FigureS1 that illustrates the survival curve for TCGA breast cancer patients

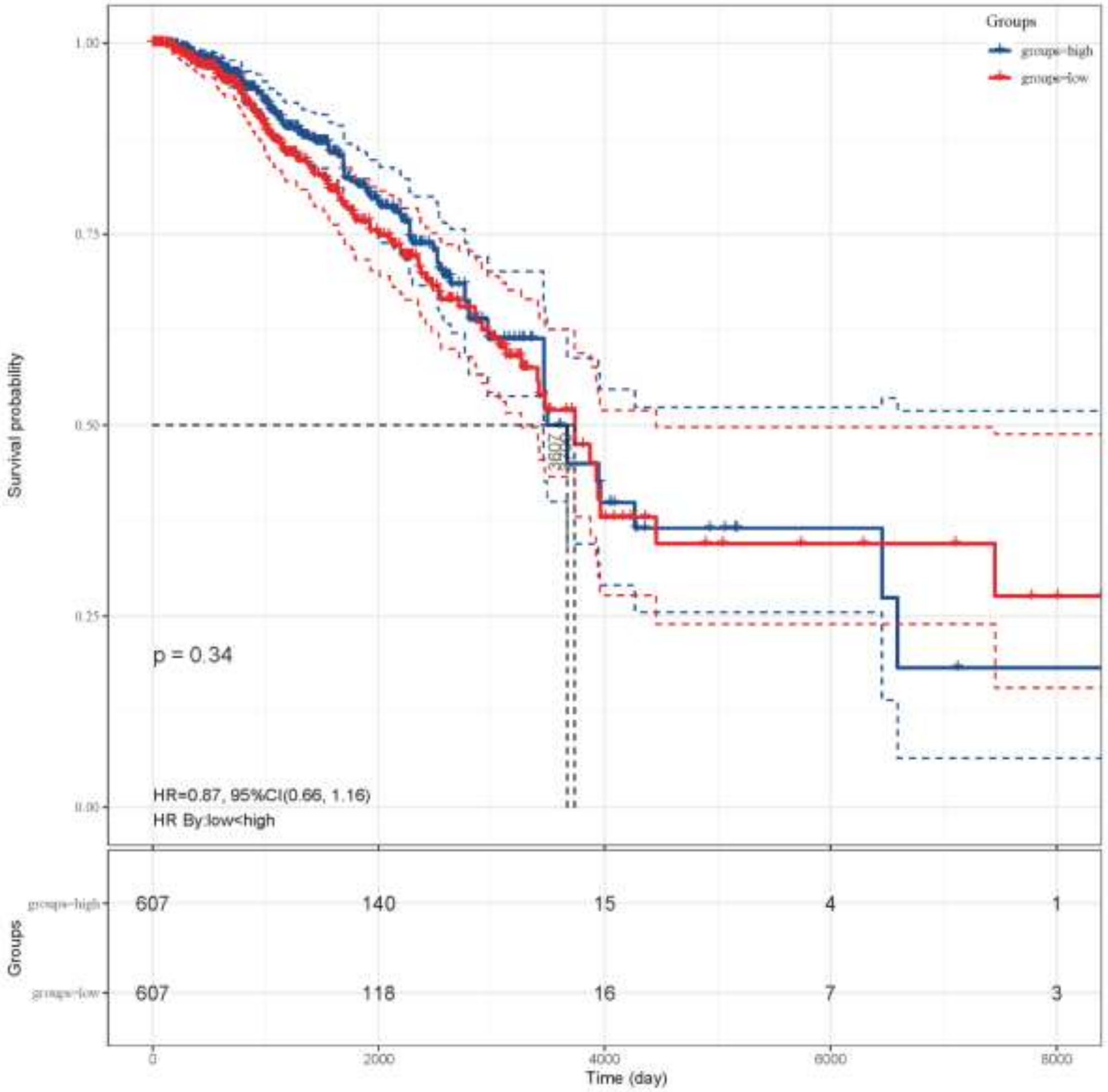

Supplement: Supplementary file 4 [file medi-102-e34271-s004.pdf]
